# Land-use-associated stressors interact to reduce bumblebee health at the individual and colony level

**DOI:** 10.1098/rspb.2023.1322

**Published:** 2023-10-11

**Authors:** Florian Straub, Markus Birkenbach, Sara D. Leonhardt, Fabian A. Ruedenauer, Jonas Kuppler, Lena Wilfert, Manfred Ayasse

**Affiliations:** ^1^ Institute of Evolutionary Ecology and Conservation Genomics, Ulm University, Albert-Einstein-Allee 11, 89081 Ulm, Germany; ^2^ Plant-Insect-Interactions, Research Department Life Science Systems, Technical University of Munich, Hans-Carl-von-Carlowitz-Platz 2, 85354 Freising, Germany

**Keywords:** anthropogenic influence, bumblebee, nutrition, neonicotinoid, pollinator health

## Abstract

In agricultural landscapes, bees face a variety of stressors, including insecticides and poor-quality food. Although both stressors individually have been shown to affect bumblebee health negatively, few studies have focused on stressor interactions, a scenario expected in intensively used agricultural landscapes. Using the bumblebee *Bombus terrestris*, a key pollinator in agricultural landscapes, we conducted a fully factorial laboratory experiment starting at nest initiation. We assessed the effects of food quality and insecticides, alone and in interaction, on health traits at various levels, some of which have been rarely studied. Pollen with a diluted nutrient content (low quality) reduced ovary size and delayed colony development. Wing asymmetry, indicating developmental stress, was increased during insecticide exposure and interactions with poor food, whereas both stressors reduced body size. Both stressors and their interaction changed the workers’ chemical profile and reduced worker interactions and the immune response. Our findings suggest that insecticides combined with nutritional stress reduce bumblebee health at the individual and colony levels, thus possibly affecting colony performance, such as development and reproduction, and the stability of plant–pollinator networks. The synergistic effects highlight the need of combining stressors in risk assessments and when studying the complex effects of anthropogenic stressors on health outcomes.

## 1. Introduction

Insects have faced severe losses globally during the past few decades [1,2]. Insect biomass, species numbers and diversity are declining alarmingly, as highlighted in recent studies [1,3,4]. Declines in pollinating insects are of particular concern, since such insects play an important functional role in the ecosystem by pollinating the majority of crops and wild flowering plants [5,6]. Thereby, they are essential for maintaining sustainable food security, for ensuring stable communities between plants and their associated animals and, hence, for maintaining biodiversity [5,7,8]. Among the pollinators, solitary and social bees stand out not only for being one of the most diverse groups [9] but also for their pollination services [7]; they are also being especially badly affected by species declines [10]. Bumblebees play a particular role in temperate regions, being active at low temperatures and pollinating crops more efficiently than honeybees or other wild bees [5]. As they are social insects, they can reach high population densities and are typically among the major pollinator species throughout the whole flowering season. Like all wild bees, they rely on floral resources, i.e. nectar and pollen, the latter being the only food source for larvae, which makes them particularly vulnerable to limited floral resources [11]. Various factors, such as climate change, invasive species or pathogens, are resulting in bee declines and are also affecting their health [2,10]. Habitat loss, pesticides and monotonous diets, which all are closely linked to land-use, have been identified as key drivers for these declines [6,10,12]. Whereas pollinators face all these stressors simultaneously in nature, their effects are however generally tested separately, including environmental risk assessments for pesticides [13].

Agriculturally managed landscapes dominate large areas worldwide [14] resulting in a decrease in floral diversity and abundance [15], which in turn, reduces bee nutrition [16,17]. Food quality and quantity play a major role in the development and physiology of larvae [17,18] and determine body size in wild bees [19,20]. Nutritional shortages in the form of low-quality food impair reproductive success [21], and alter susceptibility to diseases and infections in honeybees and bumblebees [22]. In nature, lowest pollen nutrient concentration in plants was estimated around 2.5% of total protein with high variation among plant species [23]. To meet their nutritional requirements, bumblebees continuously assess pollen quality during foraging and select high-quality food, with a preference for pollen that is rich in protein and low in fat [24–26]. Large crop fields or monocultures provide large amounts of resources, but only a narrow range of nutrients, which hinders bees from self-selecting resources that meet their nutrient requirements [17]. Furthermore, such areas do not provide resources outside the flowering period.

In order to increase agricultural yields, pesticides are frequently used, often with unintended detrimental effects on beneficial insects. Neonicotinoids are a class of insecticides introduced into farming practice in the 1990s [27]. Experiments use field-realistic doses to simulate pesticide exposures to which bees are exposed in nature. Field-realistic doses of neonicotinoids have been shown to reduce growth and reproduction in bumblebee colonies [21]. In the solitary bee *Osmia bicornis,* field-realistic doses of clothianidin decreased antennal sensitivity [28] and reduced mating success of males because of a disruption of pheromones [29]. Furthermore, imidacloprid and clothianidin impaired behavioural patterns, such as foraging in solitary bees [28] and bumblebees [30], and memory and learning in bumblebees [31]. Moreover, clothianidin increased infections by suppressing the bee immune system [32,33]. Because of these adverse effects on pollinators and especially on bees, the use of neonicotinoids has been severely limited in the EU since 2018 [27]. Currently, acetamiprid is the only neonicotinoid that can be applied without restrictions [34,35]. It is used in many bee-attractive crop plants, e.g. in rapeseed against the rape pollen beetle and further pest insects such as aphids and fruit flies in various amounts ranging from 125 to 600 g ha^−1^ [36]. Contrary to several other neonicotinoids, which are banned now, acetamiprid is supposed to be bee-safe because of its lower toxicity [37]. We have therefore used this neonicotinoid in our experiments. Moreover, sub-lethal doses of acetamiprid have recently been shown to negatively affect bumblebees, causing neuromuscular disfunctions [38] and reducing the production of larvae and workers in the field [39].

Experiments testing the effects of pesticides on bumblebees are usually conducted on individuals in small queen-less microcolonies rather than in full colonies with a queen (queen-right colonies) and focus on simple developmental and reproductive traits [40]. Although such simple assays are considered as an effective tool in pesticide risk assessments [41], they reflect neither the life history of bees with effects on larvae, pupae and adult workers, nor the stressors encountered in the field. Growing evidence is now questioning the reliability of experiments in queen-less microcolonies and has even shown contradicting effects in queen-less and queen-right colonies [42]. Although other stressors such as nutritional stress are usually excluded, bees are challenged by a plethora of stressors in the field and, thus, investigations on a single stressor may not represent real-world field situations. Indeed, recent studies using laboratory and semi-field set-ups have demonstrated that the combined effects of nutritional stress and insecticides exacerbate each other, while high-quality diet buffers and mitigates the effects of insecticides in honeybees [43,44], bumblebees [45–47] and solitary bees [48,49].

The increasing use of pesticides and declining food resources, mainly attributable to intensive land management, are considered to be key environmental factors affecting pollinator health in nature [16]. As defined by López-Uribe *et al*. [50], pollinator health in social species results from a multilevel interaction of various traits at the individual and colony levels, ultimately affecting population health and, as such, needs to be assessed at multiple levels. This integrative approach combines diverse traits related to reproduction, body size or physiology, all of which play important roles in colony performance of social insects. For example, the cuticular chemical profile of bumblebees fulfils a key role in intracolonial communication, such as task allocation and suppression of worker reproduction [51,52], and individual body size is correlated to foraging behaviour [53,54] and thermoregulation [55]. Extending this approach to physiological traits, the immune response of individual bees, for example, is an important factor for colony health, identifying and eliminating potential pathogens and preventing their spread to other nest-mates. Although all of these traits are measured at the individual level, they are connected at the colony level, as they might directly affect colony performance.

Here, we have attempted to reflect real-world conditions by investigating the effects of food quality and insecticide exposure, individually and in combination, on bumblebee health. We aimed to answer how food quality and/or insecticide exposure and their interaction are affecting bee health and which health traits are affected in particular. Therefore, we have studied individual- and colony-level health parameters in queen-right colonies starting with nest initiation, as population health can be affected by deleterious effects on different levels of organization in these social insects. Moreover, bumblebee population health might in turn affect pollination and disrupt stable plant–pollinator interaction networks, since bees pollinate many crops and wild flowers.

## 2. Material and methods

### (a) Study species

We used *Bombus terrestris* (LINNAEUS, 1758) in all experiments. The founding queens for all experimental colonies were obtained from Koppert (Koppert Biological Systems, The Netherlands). Colonies were reared in transparent plastic boxes (18 × 17 × 7 cm^3^; electronic supplementary material, figure S1). For colony initiation, two overwintered queens were placed together to stimulate breeding and egg laying [56]. One queen died during the first week and was removed immediately after death. Colonies were kept in constant darkness at a room temperature of 27 ± 2°C and a relative humidity of 60–70% in an insect rearing room.

### (b) Overview of experimental design

We tested the direct and combined effects of nutritional stress (pollen quality) and insecticide exposure in a fully factorially designed experiment. Queen-right bumblebee colonies were randomly assigned to one of the four treatments and either treated with (a) high-quality pollen and no insecticide as a control (group 1, 6 colonies), (b) low-quality pollen without insecticide (group 2, 7 colonies), (c) high-quality pollen and insecticide (group 3, 7 colonies) or (d) a combination of low-quality pollen and insecticide (group 4, 9 colonies). For insecticide exposure, we used the neonicotinoid acetamiprid. Bumblebees could feed ad libitum on sugar solution (with or without pesticide) and pollen (high- or low-quality pollen, with or without pesticide), depending on the treatment group to which they had been assigned. Workers were colour-coded several times a week after emergence and divided into cohorts based on their age (see electronic supplementary material, table S1, on how cohorts were represented in each batch). Each colony was maintained until it had reached 45 workers. All individuals were then individually freeze-killed in liquid nitrogen and processed according to their batch, in which various individual-level health parameters were measured, as certain measurements could not be conducted in parallel on the same individuals (electronic supplementary material, figure S2 and table S2; see below for details). Thus, all health parameters were measured in a subsample of bees of each colony. In the first cohort batch (B1—encapsulation, *n* = 339, 11–12 bees per colony), bumblebees were between 6 and 14 days old (mean 7.5 days), whereas the second cohort batch (B2—chemical analysis and ovary size, *n* = 289, 9–10 bees per colony) contained individuals of 10 to 17 days of age (mean 11.6 days). Bumblebees in the third cohort batch (B3—fat body analysis, *n* = 289, 9–10 bees per colony) were the oldest and were aged between 13 and 25 days (mean 15.9 days). Body size and wing asymmetry, which had previously been determined during larval development, were measured in individuals of B1 and B2 (*n* = 625, 20–22 bees per colony) to maximize the sample size [57].

#### (i) Insecticide application

Bumblebees were fed pollen and nectar treated with 5 ng acetamiprid ((*E*)-N1-[(6-chloro-3-pyridyl)methyl]-N2-cyano-N1-methylacetamidine) per gram reflecting a field-realistic dose as found in oilseed rape [34]. We used the pure substance instead of a commercially available formulation to avoid any side effects of co-formulants. For the application in bumblebee colonies, 5 µg ml^−1^ acetamiprid (greater than 98%, Pestanal, Sigma-Aldrich, Hamburg, Germany; dissolved in demineralized water) was diluted in a 50% sugar solution, made from 73% API-Invert (Suedzucker AG, Mannheim, Germany; diluted in water) or was added to the respective pollen mixtures (for preparation see below).

#### (ii) Pollen diet preparation

Both pollen mixtures were prepared with honeybee-collected polyfloral pollen (hives located in Blaustein, Baden-Wurttemberg, Germany). Instead of cellulose, which is often used to reduce pollen quality but causes high mortality rates in larvae [18], we used pollen exines. Exines have no adverse effect on bees and reduce the overall nutrient content (dilution of protein content) whereas use of mono- versus polyfloral pollen only results in differing nutrient compositions and ratios, which could be detrimental (table 1) [18]. Fatty acid concentration was not affected in the exines, although amino acid (protein) concentrations and the amino acid to fatty acid ratio in the diets were reduced. To produce pollen exines, we followed the methods by Tainsh *et al*. [18] (see electronic supplementary material for further details). High-quality pollen was mixed with pollen exines at a ratio of 7:3 (v/v) to reduce quality, with pure API-Invert (180 ml per litre of pollen) being added to prepare the pollen portions. Fatty acid concentration reflected the mean values of fatty acids, while amino acid content in the low-quality treatment was slightly above the minimum (2.5%) found in natural pollen [23].

**Table 1.** Nutritional properties of high- and low-quality pollen. Mean total amount ± SD (µg mg^−1^) of amino acids and fatty acids and their ratio (for further details, see electronic supplementary material, tables S3 and S4).

	high-quality	low-quality
amino acid (AA)	71.93 ± 9.27	37.09 ± 21.16
fatty acid (FA)	56.49 ± 12.46	56.65 ± 8.73
ratio AA:FA	1.27 ± 0.74	0.65 ± 2.42

### (c) Bumblebee health parameters

We measured food consumption, development and worker interactions at the colony level. At the individual level we assessed encapsulation response, cuticular chemical profile, ovarian activation, body size and fat body content.

#### (i) Colony development and worker behaviour

Pollen portions and sugar water containers were weighed every 2 days to calculate food consumption. Pollen portions were replaced every two days with portion sizes adapted to previous consumption in order to assure ad libitum conditions. Sugar water containers were refilled when necessary. We recorded food consumption during the experiment to check that bees ingested food and that food resources were always available ad libitum. Reference samples for pollen and sugar water placed in a similar set-up were also weighed to correct for evaporation. To measure colony development, we recorded the hatching date of individual workers and the number of workers per colony.

In each colony, we video-taped (Panasonic HC-X810) interactions between individual workers of the first brood (maximum of 12 workers) three times under constant red light for 10 min, after at least three, six or nine workers had hatched (in total 56 observations), because interactions can provide information about the social structure in the colony. Using the observation software The Observer XT (v. 15.0.1200, Noldus Information Technology, The Netherlands), we analysed the number of different interactions related to dominance behaviour in the nest. We distinguished between (i) nudging, defined as a fast movement forwards with direct face contact of the bees, (ii) backing behaviour, a fast movement backwards by the nudged bee [58] and (iii) antennation between workers without nudging or backing.

#### (ii) Encapsulation response

Worker bees from batch B1 (*n* = 339, 29 colonies) were used for an encapsulation assay, using standard protocols for bumblebees [54,59]. We employed the encapsulation response as a proxy for an immune response, because it mimics, for example, the eggs of parasitoid conopid flies [60]. Workers were anaesthetized in crushed ice for at least 30 min before a piece of nylon thread (approx. 2 mm length) was implanted in the abdomen via the intersegmental membrane. Melanization was stopped after 2 h by freeze-killing bumblebees in liquid nitrogen. The nylon threads were retrieved from the bees and mounted on microscopic glass slides and photographed using an Axiocam 208c microscope camera (Zeiss, Germany) attached to a Stemi 508 stereo microscope (Zeiss, Germany). To assess the degree of melanization, the mean grey value of each implant was measured using ImageJ software.

#### (iii) Cuticle surface extracts and chemical analysis

We analysed the cuticular chemical profiles of workers because of its key role in intracolonial communication [51]. Bees from batch B2 (*n* = 289, 29 colonies) were thawed at room temperature for 4 min and rinsed in 1 ml *n*-pentane (SupraSolv, 99.9%, Supelco) for 1 min to extract cuticular chemical compounds. The extracts were concentrated to a final volume of 500 µl under a gentle nitrogen stream. We then added 10 µl of an internal standard (dodecane; 99%, Sigma, Germany, 100 µg ml^−1^ in *n*-pentane) for quantitative analysis. Samples were stored at −20°C. Chemical analyses were performed by means of a gas chromatograph (Agilent 7890A, Agilent Technologies, Waldbronn, Germany) with a DB-5 capillary column (30 m × 0.25 mm inner diameter, J&W) and a flame ionization detector. Hydrogen was used as a carrier gas at a constant flow of 2.0 ml min^−1^. One microlitre of extract was injected splitless into the gas chromatograph at an injector port temperature of 310°C. After an initial time of 1 min at 50°C, the splitter was opened and the oven temperature increased continuously by 10°C min^−1^ to a final temperature of 310°C at which temperature was held for 35 min, resulting in a total run time of 62 min. Compounds were identified based on analyses of reference substances and earlier studies [52,61].

#### (iv) Ovarian development

Workers from batch B2 were also used to measure the reproductive state of bumblebees. Each bee was dissected and the terminal oocyte size of the most developed ovary was measured using the analytical software ZEN (version 3.2, blue edition, Zeiss). Contrary to honeybees, worker bumblebees are not sterile and compete with queens in laying unfertilized eggs, which result in normal, functional haploid males, during the competition phase [62].

#### (v) Body size

As a proxy for body size, we used the centroid size (CS) of forewings which was shown to be a reliable measure in several species, among them *B. terrestris* [63]. The forewings of each individual worker (batches B1 and B2, *n* = 625, 29 colonies) were cut off and mounted between two microscopic glass slides. Wings were digitized using a microscope camera attached to a stereo microscope. On each wing we set a total of 18 landmarks (electronic supplementary material, figure S3) by using tpsDIG (v. 2.31, available at https://sbmorphometrics.org/). By means of MorphoJ software (v. 1.07a, available at https://morphometrics.uk/MorphoJ_page.html), we calculated CS for each individual, following Klingenberg [63]. Furthermore, wing asymmetry, a measure for developmental stability, was calculated using CS of left and right wings of each individual with the following formula [57]:

$$\mathrm{Wing}\;\mathrm{asymmetry}=\frac{\left|{\mathrm{CS}}_{\mathrm{right}}\;-CS_{\mathrm{left}}\right|}{({\mathrm{CS}}_{\mathrm{right}}\;+CS_{\mathrm{left}})/2}.$$

#### (vi) Fat body analysis

Whereas queens use the fat body as an energy source for hibernation, it provides workers with energy to maintain immune responses during starvation times [64,65]. We used Folch's method to obtain absolute fat body mass, with drying days and washing steps being adapted to our needs [66]. We weighed initial wet mass and dry mass of bisected abdomens (stored in a glass vial) of bumblebees from batch B3 (*n* = 289, 29 colonies) after 4 days at 70°C. Subsequently, 1 ml Folch's reagent (chloroform:methanol 2 : 1 v/v) was added and replaced every day for four consecutive days. Folch's reagent was finally removed before the drying process was repeated at 70°C for 4 days and the final dry mass was weighed. We calculated the relative proportion of fat by using the absolute fat body mass (subtracting the final dry mass from the initial dry mass) divided by the initial dry mass to correct for differences in body size.

### (d) Statistical analysis

All statistical analyses were performed in R (v. 4.2.0) [67] with food quality and insecticide as fixed factors. To analyse pollen and nectar consumption, we calculated generalized additive models (GAMs) by using the *gam* function from the *mgcv* package (v. 1.8.40) [68]. Furthermore, we calculated linear mixed-effect models (LMEs) by using the *lmer* function from the *lme4* package (v. 1.1.29) [69] with the Kenward–Roger approximation (lmerTest package, v. 3.1–3 [70]) and generalized linear models (GLMs) by using the *glm* function from the basic R package *stats*. Total amount of chemical compounds per individual was standardized by body size. Because of zero-inflated data in ovary size, we calculated generalized linear mixed models (GLMMs) with the *glmmTMB* function from the *glmmTMB* package (v. 1.1.4) [71] with a truncated Poisson distribution. Whenever we found interactive effects of food quality and insecticide exposure, we ran *post hoc* tests by using the *emmeans* function from the *emmeans* package (v. 1.7.4-1) [72]. Additionally, we extracted model estimates with confidence intervals for all measured traits. Depending on the respective health trait, we set colony, cohort and observation as random factors (for more statistical details, see electronic supplementary material, table S5). All model assumptions were validated using the *check_model* function from the *performance* package (v. 0.9.0) [73] and were adequate.

To analyse differences in chemical profiles, we calculated absolute amounts of each chemical compound based on the peak area of the internal standard, which was summed to total amount of cuticular compounds. We then analysed the relative proportions of compounds in the chemical profile using PRIMER 6 software (v. 6.1.15) with the permutational multivariate analysis of variance + (PERMANOVA+) add-on (v. 1.0.5). Permutational analyses PERMANOVA (9999 permutations, permutation of residuals under a reduced model, Type III partial sums of squares, square root transformation, Bray–Curtis similarity distance matrix), which we used to compare the ratios of compounds in the cuticular profiles of bumblebees, is a method widely used in ecology and that simultaneously tests the response of multiple variables to several factors without the strict assumptions of an ANOVA (e.g. normality) [74]. In both analyses, namely cuticular chemical profile and the total amount, we included food quality, insecticide and oocyte size as fixed factors and colony and cohort as random factor. We included oocyte size as this trait is known to correlate with the chemical profiles of bumblebees [61].

## 3. Results

### (a) Colony development

Food consumption changed as colonies developed. Pollen consumption significantly increased over time (days after treatment start; GAM: ED = 57.3%, *n* = 763, *F* = 115.347, *p* < 0.001; electronic supplementary material, figure S4A) and nectar consumption increased with increasing number of workers (GAM: ED = 74.1%, *n* = 399, *F* = 168.580, *p* < 0.001; electronic supplementary material, figure S4B). We included days after treatment start as an additional factor in the model for pollen consumption. Because mainly workers feed on nectar, we started the analysis of nectar consumption with the hatching of the first worker and included the number of workers as a factor. Neither food quality nor insecticide exposure affected pollen or nectar consumption. However, a trend was seen for an interaction between food quality and insecticide exposure leading to an increase in pollen consumption (GAM: *t* = 1.705, *p* = 0.089) and a decrease in nectar consumption (GAM: *t* = 1.678, *p* = 0.094).

**Figure 1.**
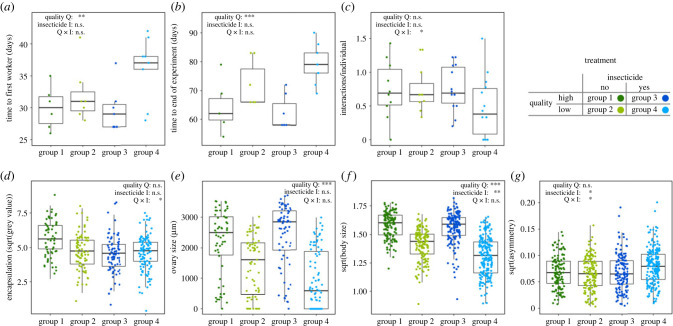
Effects of food quality and insecticide exposure on health traits of *Bombus terrestris* colonies (*a–c*) and individual health traits (*d–g*). (*a*) Time until the first worker hatched. (*b*) Time until the experiment was terminated as 45 workers hatched. (*c*) Sum of all recorded worker interactions (nudging, backing, further interaction with antennation) standardized by number of workers per colony. (*d*) Encapsulation response measured as grey scale values of implants. (*e*) Size of the terminal oocyte in each bumblebee ovary. (*f*) Centroid size as a proxy for body size, based on 18 landmarks on forewings. (*g*) Wing asymmetry between left and right forewings, based on centroid size of 18 landmarks. Colonies were fed with differing food qualities (high quality or low quality) and either were exposed to acetamiprid (yes) or were not exposed (no) according to the respective treatment group. Asterisks indicate significant differences of the respective treatment (n.s. = not significant; *p < 0.05, **p < 0.01, ***p < 0.001).

Hatching time of workers (time until the first worker hatched) decreased by around 5 days with high-quality food (LMM: *χ*^2^ = 8.834, *n* = 29, *p* = 0.003; figure 1*a*; model estimates electronic supplementary material, figure S5A) and was not affected by insecticide exposure or an interaction (electronic supplementary material, table S6). The same results were found for the time until the experiment was terminated (45 workers). The duration of the experiment differed significantly (around 14 days) in relation to food quality (LMM: *χ*^2^ = 30.540, *n* = 29, *p* < 0.001; figure 1*b*; model estimates electronic supplementary material, figure S5B) but not with insecticide treatment or with the interaction. No difference between treatments was detected in the number of workers of the first brood (electronic supplementary material, table S6).

### (b) Bumblebee behaviour

The social behaviour of workers (nudging, backing, antennation) of the first brood batch was affected neither by food quality nor insecticide exposure. Food quality and insecticide exposure also had no effect when all three behavioural categories were summed into total interactions to represent the activity level. We found an interactive effect of food quality and insecticide decreasing the activity level (LMM: *F* = 5.759, *n* = 52, *p* = 0.021; figure 1*c*; model estimates electronic supplementary material, figure S5C; see electronic supplementary material, table S8, for *post hoc* tests).

### (c) Encapsulation response

Insecticide exposure together with food quality significantly reduced the immune response towards the implant (LMM: *F* = 6.602, *n* = 289, *p* = 0.017; figure 1*d*; model estimates electronic supplementary material, figure S5D; see electronic supplementary material, table S8, for *post hoc* tests). Furthermore, we found a trend of insecticide treatment affecting the encapsulation response (LMM: *F* = 3.966, *n* = 289, *p* = 0.059).

### (d) Cuticular chemical profile and total amount of compounds

**Figure 2.**
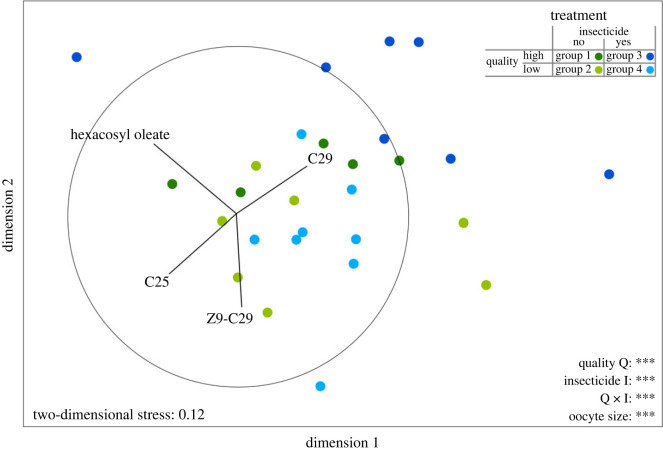
Cuticular chemical profile displayed in two dimensions following non-metric multidimensional scaling (NMDS) based on Bray–Curtis similarities of the relative amounts of compounds. The graph shows the cuticular chemical profiles of workers of four different treatment groups. Every coloured dot represents the average chemical profile per colony. Vectors visualize the compounds that contributed most in the dissimilarity of bouquets (C25 = pentacosane, Z9-C29 = (*Z*)-9-nonacosene, C29 = nonacosane). Colonies were fed with differing food qualities (high quality or low quality) and either were exposed to acetamiprid (yes) or were not exposed (no) according to the respective treatment group. Asterisks indicate significant differences of the respective factor on the chemical profile (***p < 0.001).

We analysed and identified a total of 58 chemical compounds (electronic supplementary material, figure S6 and table S7) that had been identified in earlier studies [52,61]. Our multivariate analysis showed that the cuticular chemical profiles of bumblebees differed between food quality (PERMANOVA_Quality_: Pseudo-*F*_1,285_ = 25.682, *n* = 289, *p* < 0.001; figure 2), insecticide exposure (PERMANOVA_Insecticide_: Pseudo-*F*_1,285_ = 7.730, *n* = 289, *p* < 0.001) and oocyte size (PERMANOVA_Ovary_: Pseudo-*F*_1,285_ = 24.240, *n* = 289, *p* < 0.001). Furthermore, we found an interactive effect of food quality and insecticide exposure on the chemical profile (PERMANOVA_Q x I_: Pseudo-*F*_1,285_ = 6.702, *n* = 289, *p* < 0.001; see electronic supplementary material, table S8, for *post hoc* tests). We performed SIMPER analysis to find the chemical compounds that explained the main differences of bouquets between the four treatments and found the *n*-alkanes pentacosane (C25) and nonacosane (C29), the alkene (*Z*)-9-nonacosene (Z9-C29) and the wax-type ester hexacosyl oleate explained at least 20% of the difference.

Total amount of cuticular chemical compounds was affected neither by food quality nor insecticide nor by an interaction of both. Higher total amount was exclusively explained by increasing ovary size (LMM: *F* = 8.688, *n* = 283, *p* < 0.001).

### (e) Ovarian activation

Decreasing ovary size of bumblebee workers was explained by food quality (GLMM: *χ*^2^ = 15.212, *n* = 289, *p* < 0.001; figure 1*e*; model estimates electronic supplementary material, figure S5E), but not by insecticide exposure nor by an interaction. In both groups with low-quality pollen, we observed undeveloped ovaries (stage 0, according to Duchateau & Velthuis [62]) whereas high-quality pollen groups always had visible oocytes.

### (f) Body size and asymmetry

Food quality and insecticide exposure both decreased the body size of workers, which ranged from 0.887 mm to 1.817 mm (LMM_Quality_: *F* = 86.206, *n* = 625, *p* < 0.001; LMM_Insecticide_: *F* = 9.362, *n* = 625, *p* < 0.01; figure 1*f*; model estimates electronic supplementary material, figure S5F); additionally, a trend was noted for an interactive effect (LMM: *F* = 3.611, *n* = 622, *p* = 0.069).

Wing asymmetry increased with insecticide exposure (LMM: *F* = 5.531, *n* = 625, *p* = 0.027; figure 1*g*; model estimates electronic supplementary material, figure S5G) and with an interactive effect of insecticide and food quality (LMM: *F* = 5.473, *n* = 625, *p* = 0.027; see electronic supplementary material, table S8, for *post hoc* tests).

### (g) Fat body

Fat body was not affected by any of the treatments in our experiment. Neither food quality nor insecticide exposure nor an interaction between the two treatments affected the fat body content of worker bumblebees (electronic supplementary material, table S6).

## 4. Discussion

Both stressors, food quality and insecticide exposure, affected various health traits at the colony and individual level in *Bombus terrestris* (summarized in electronic supplementary material, table S6). We found that some health traits were affected by food quality alone, whereas others were affected by an interaction or both stressors. Food quality affected colony developmental time and oocyte size in individual workers. All further traits, except for fat body content and number of workers in the first brood which were not influenced, were affected either by both stressors and/or their interaction. Although acetamiprid has a manifold lower toxicity compared to other neonicotinoids and is supposed to be bee-safe, we could find effects on health traits, clearly attributable to acetamiprid. Reduced bee health can be expected to affect colony-level traits and colony success directly, and would therefore translate into effects on pollinator health at the population level in the field and on pollination as a whole [50].

### (a) Diet and insecticide affect individual physiological processes

We did not detect any treatment-related differences in feeding behaviour, while bees in previous studies consumed less food when supplemented with pollen [44,75]. However, bumblebees in our study did not alter feeding behaviour in any treatment and differences in food consumption were explained by time (pollen consumption) and the number of workers (nectar consumption). In growing and developing colonies, larvae require pollen and workers use nectar as their energy source [11]. This causes an increase in food consumption over time and shows a strong correlation with colony size. Our low-quality pollen comprises a dilution of proteins compared with the high-quality pollen diet, whereas fatty acid concentration stays the same. Because bumblebees assess food quality and prefer high-quality pollen [25], we would have expected an increase in food consumption in the low-quality treatments. However, Ruedenauer *et al*. [76] have found that *B. terrestris*, when assessing pollen quality, rely primarily on pollen fatty acid content, which is not affected by the exines (table 1). Thus, bumblebees in our experiment might have used differing amino acid to fatty acid ratios or the fatty acid concentration alone to avoid excessive feeding on low-quality pollen. Excessive feeding would have resulted in an unbalanced diet with too much fat and would have been detrimental [76]. Thus, diverse landscapes with abundant high-quality resources are crucial for bee health and enables them to cover nutritional needs continuously, without the risk of ingesting an unbalanced diet [77].

The fat body content of workers was affected by neither food quality nor insecticide exposure or an interaction. Usually, queens have large fat body stores to survive hibernation [64], whereas workers do not hibernate and thus have no such need. However, the fat body in workers provides energy in starvation periods and helps to maintain immune responses [64,65]. Bees may try to retain a certain level of fat, even under stressful conditions, to provide this energy during harsh times. Additionally, the fat body in our workers was potentially so small that it is not affected by food quality and we could not detect differences, as also shown in a recent study on amino acid consumption in bumblebees [78]. Furthermore, an investment in immediate traits such as the immune response instead of reserves might be advantageous during nutritional shortages. In social insects, investment in immunity is important at the colony level, as good immunity will prevent possible infections from spreading in the colony.

All individuals showed an encapsulation response, although the response levels differed. The combined treatment reduced the level of melanization and a trend of the insecticide treatment alone, with higher levels of melanization indicating a stronger immune response [59]. The immune system identified and melanized the implants but the stressors might have weakened the encapsulation response. The implants simulate an antigen and challenged the immune system, similar to the eggs of parasitoid conopid flies, which are frequently found in bumblebees [60]. Neonicotinoids are known to downregulate the immune response in honeybees and increase susceptibility to diseases [32,33]. Moreover, a protein-deficient diet reduces the expression of immune genes [79]. Thus, the combination of low-quality food, which reduces melanization, and the neonicotinoid treatment, which has been suggested to downregulate the immune response, causes lower encapsulation responses in our experiment. These experimental results suggest that bumblebees foraging in landscapes with high land-use intensity and that are thus faced with poor-quality food and constant changes of pesticide exposure will be more prone to pathogens and parasites, which might further impact their populations and drive insect declines as suggested in recent studies [80,81]. Bumblebees are eusocial insects that show division of labour and overlapping generations [5,51], both of which make an investment in immune responses at the individual level to combat parasites or pathogens that affect colony-level health. This investment prevents the spread of disease and subsequent reductions in the workforce, and is thus vital for securing stable bumblebee communities in the landscape context.

Ovarian activation in our experiments is affected exclusively by food quality. Since dietary protein is required not only for melanization, but also for oogenesis in bumblebees [62], a lack of protein might have resulted in undeveloped ovaries and smaller oocytes. Our low-quality pollen only contained around 50% of the original protein content (table 1) supporting the hypothesis that a lack of specific nutrients (i.e. protein) causes reduced oocyte size. In bumblebee colonies, usually the queens reproduce. By contrast to honeybees, female workers can however also produce males if the queen's reproductive control wanes during the competition phase [62]. Since workers do not produce queens, they are not directly involved in the reproductive output of their own colony. However, if the queen dies prematurely, worker-laid eggs develop into males that, after leaving their colony, can fertilize virgin queens, thereby contributing to the stabilization of bumblebee communities. We did not test queens in our experiments, but if queens are affected similarly to workers, the queen might also be negatively affected by low-quality pollen and thus have a strong impact on colony performance under natural conditions.

### (b) Stress-induced changes in the chemical surface profile of *B. terrestris*

In social insects, cuticular lipids have a key function in intra-nest communication and task allocation [51]. The chemical surface profiles of workers in our experiment were affected by all treatments highlighting the susceptibility of the chemical profile to stressors. Our results are also in agreement with those of our previous field study showing that land-use intensity is correlated with changes in the chemical profile of the bumblebee *B. lapidarius* [12], suggesting that these changes might also be attributable to food quality and insecticides. Alterations in the chemical profile might be explained by a lack of nutrients that are required for the biosynthesis of cuticular lipids. Insects synthesize hydrocarbons by elongating precursor compounds derived from their diet [82] and, hence, differences in food quality and quantity might result in changes to the chemical profile. Insecticide-induced changes in the chemical profiles might be the result of a disruption of biosynthetic pathways, similar to a downregulation of the immune system. Indeed, pesticides have been shown to change the chemical profile and to disrupt mating in solitary bees [29]. Furthermore, the chemical profile and the total amount of chemical compounds in our experiment are affected by ovary size, a result that agrees with those of earlier studies [61]. The cuticular chemical profile plays an important role in intra-nest communication, and compositional changes in the profile might directly affect intra-colonial behaviour and thus overall colony communication and performance.

The four compounds (pentacosane, nonacosane, (*Z*)-9-nonacosene and hexacosyloleate) that contributed most to the variation of chemical profiles between the four treatment groups are known to play important roles in task allocation of bumblebees and as components of fertility signals [61,83]. The cuticular chemical profiles of queens combined with dominance behaviour prevent workers from laying unfertilized eggs, with compounds such as pentacosane being of crucial importance [84,85]. Pentacosane increases rapidly in workers shortly before the competition point, when queens lose their dominance and workers start to lay unfertilized eggs [61,83]. An early loss of the queen's dominance including reproduction can have dramatic effects on colony maintenance, especially in early stages of colony development when the queen has not yet laid eggs from which young queens hatch. However, this effect is not regulated exclusively by individual substances, but by the chemical profile as a whole. Even though queen dominance might be reduced under land-use-induced stress, the workers might not be able to respond to it because of poor resources, since they also show undeveloped ovaries in our experiments. Some evidence for the increase of pentacosane in relation to land-use intensity has been presented in a recent study [12] and supports the idea that stressors such as food quality and pesticides may affect changes in cuticular pheromones in bumblebees and thus influence colony success in total.

### (c) Stress affects individual-level and colony-level development

Low-quality pollen reduced the body size of workers, which may be due to a lack of nutrients essential for their larval development. Several studies have shown that low-quality pollen and low pollen diversity result in a decrease in body size in bumblebees [21,22] and solitary bees [19,20]. Bumblebees assess pollen quality to optimize foraging [25,26], likely also to prevent a decrease in body size of nestmates. Smaller bees have previously been shown to forage less [53] and a smaller body size is negatively correlated with survival under stressful conditions [86]. Foraging, on the one hand, is related to the food provision of the colony, with bees foraging in poor-quality landscapes spending more energy and providing less food to their colony, and, on the other hand, is correlated to pollination. Bees with lower foraging activity also pollinate fewer flowers and thus affect the reproductive output of plants. Furthermore, we have shown that insecticide exposure reduced body size (here wing size) and increased wing asymmetry (a measure of developmental stability), both of which are connected to larval development [63], while a recent study using specimens from museums could link increasing wing asymmetry to climatic conditions such as increasing temperature [87]. Thus, acetamiprid and a lack of nutrients affect bumblebee larval development and cause a decrease in body size.

In our study, experimental colonies feeding on low-quality food showed slower development, which is attributable to a lack of nutrients. Earlier studies have suggested a correlation between pollen intake and colony development [21,88] with subsequent effects on population sizes [17]. Because of our experimental design, we did not include colony parameters such as colony size, survival or production of reproductives; however, data concerning these traits are available in the literature for various bee species (e.g. [43–49], or reviewed in [89]).

### (d) Individual health traits affect colony-level traits

We further found behavioural changes attributable to a combination of low-quality food and pesticide exposure, namely a decrease in worker interactions. Neonicotinoids have been shown to affect social behaviour in stingless bees [90], potentially by disrupting the neuronal processes that control foraging and locomotion [30] and may also affect social interactions. The decrease in interactions observed in our study might be the result of changes in chemical profiles and a disturbed communication system. However, caution is needed here, as the small number of individuals in the nest and the low number of total interactions might have affected the within-nest behaviour, as previously shown in computer simulations based on empirical data [91]. To our knowledge, our results are the first to show that food quality and insecticide exposure can affect intracolonial behaviour.

Individual health, which translates into both colony and population health [50], is often affected by poor resources and pesticides, both of which are characteristics of intensive land-use [14]. An investment in the individual immune response for instance can reduce an individual's susceptibility to pathogens or parasites thereby preventing a spread in the colony that might reduce the workforce. A reduced workforce and poorer worker health would therefore lead to a decreased food provision of the colony and ultimately would drive colony failure. Furthermore, the males resulting from worker reproduction, e.g. when a queen dies prematurely, can contribute to the colony fitness and maintaining the stability and genetic diversity of bumblebee communities. Thus, individual health amplifies over various levels, securing stable populations and communities in the landscape context. In turn, healthy pollinator populations promote pollination services, which secures crop yields and stable plant–animal networks [5,7].

## 5. Conclusion

Our results clearly show that poor conditions with low-quality food and insecticide exposure affect important health traits of *B. terrestris*. We have assessed traits at the individual and colony level in order to gain an integrated impression of the impact of stressors on bumblebee health. Decreasing pollinator health that we found in our study may affect important features linked to colony performance such as development, chemical communication, reproduction and immune response, which, in turn, may drive colony failure and decrease pollination performance with strong effects on ecosystems. Bees play a key role in the functioning of ecosystems by pollinating wild plants and important crops for human use. Thus, decreasing pollinator health might affect crop yields and cause an imbalance between plants and associated animal communities in the ecosystem, which ultimately will affect human health.

Despite its classification as bee-friendly and its low toxicity, we found effects of acetamiprid in isolation and in combination with low-quality food, which highlights the need to consider combined stressors in risk assessments, especially when studying the complex effects of anthropogenic stressors on health outcomes. When it comes to condition-dependent stressor interactions such as host–pathogen relationships, interactive effects might be unpredictable and fundamentally weakened individuals might be more susceptible to further stressors with unknown consequences. In turn, this would drive forward ongoing insect losses. Thus, diverse landscapes and a broad spectrum of host plants with low levels of pesticides are important to continuously cover the nutritional needs and fostering the health of both pollinators and other insects that rely on floral resources.

## Data Availability

All data are available in the Biodiversity Exploratories data repository BExIS (https://www.bexis.uni-jena.de/) with the accession numbers 31323 (cuticular chemical compound data), 31324 (ovary size and body fat), 31325 (encapsulation data), 31326 (body size), 31327 (interaction data) and 31328 (nectar and pollen consumption). All data are permanently archived in BExIS. Supplementary material is available online [92].
